# The Influence and Compensation Method of Eccentricity for Cylindrical Specimens in Eddy Current Displacement Measurement

**DOI:** 10.3390/s20226608

**Published:** 2020-11-18

**Authors:** Hao Zhan, Li Wang, Tingting Wang, Juntao Yu

**Affiliations:** School of Mechanical, Electrical & Information Engineering, Shandong University, Weihai 264200, China; 201916568@mail.sdu.edu.cn (H.Z.); wanglihxf@sdu.edu.cn (L.W.); wangttzh@sdu.edu.cn (T.W.)

**Keywords:** eddy current displacement sensor, eccentricity, FEM, compensation

## Abstract

The eddy current displacement sensor (ECDS) is used to realize the precise detection of the rotor radial position in the magnetic suspension motor. The eccentricity between the probe axis and the measured surface normal reduces the measurement accuracy. An ECDS mathematical model is established to analyze the influence of the measured surface curvature and eccentricity on detection results. The eddy current density distribution law of the measured surface is obtained by using the finite element method (FEM). The experimental platform is set up based on the practical engineering structure, which contains two kinds structures of the single probe and the differential. The compensation method is introduced to reduce the error caused by the eccentricity. The displacement measurement error with and without compensation are tested separately. The results show that the largest full-scale error is less than 0.8% after compensation in the single probe structure, and 0.6% in the differential structure. For the engineering application, the orthogonal direction measured value is used as the eccentricity, and the compensation order of big then small is proposed. It is thus proved that the compensation method provides a guarantee for accurate feedback and control of the rotor radial position in the magnetic suspension motor system.

## 1. Introduction

According to Faraday’s electromagnetic induction, the metal conductor in a changing magnetic field, or cutting the magnetic lines, an eddy current is generated in the target conductor. The sensor based on the eddy current effect is called the eddy current sensor. The eddy current sensor has been widely applied in the field of displacement measurement [1], defect detection [2,3], thickness measurement [4,5], and many other fields, owning to its noncontact, simple structure and high sensitivity [6,7]. Sensitivity, resolution, long-term stability, and temperature drift are usually considered when evaluating a sensor’s performance metrics [8]. The study of the eddy current sensor mostly focuses on the probe coil impedance [9,10], the optimal design for structural parameters [11,12,13], etc. The aim of these studies is to promote the eddy current sensor’s performance, such as linearity [14,15] and the range of measurement [16].

**Figure 1:**
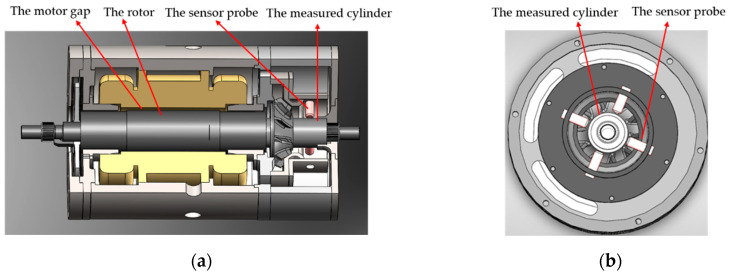
The structure graphing of the magnetic suspension motor: (**a**) the general design structure; (**b**) the rotor radial position detection unit.

Most objects measured by the eddy current displacement sensor (ECDS) are planar conductors at present. Li Y et al. presented a tilt angle model to explore the influence of tilt angle on sensor’s measured values when the measurement surface was a plane and the experiments also testified that the measuring errors can be compensated well by the Gaussian function [17]. Different surface shapes of measured conductors have different influence on the senor’s output. For three different detected surfaces—concave, plane and convexity—Zhang Y et al. built the relevant finite element models of three-dimensional eddy current field problem and the surface shape, curvature radius, and lift-off variation effects on coil’s reflected impedance had been analyzed [18]. When the surface shape is a coaxial cylindrical surface, their center axes are intersecting vertically according to the installation requirement of the ECDS. In view of the advantages of friction-free, no wear, without lubricating, no pollution, low consuming, and long life, magnetic bearings suit high or super high speed, vacuum condition, and some special conditions [19,20]. However, the eccentric problem easily occurs under the condition of high-speed rotation, which further affects the output of the ECDS. Li H et al. detected the axial displacement of maglev rotor in its radial direction through the ECDS, which employed a step surface on the rotor. Due to the limiting effect of the protective bearings, Li H thought the axial and radial displacement of the rotor are generally in the range of −1–1 mm. For a typical magnetic suspension system, the rotor radius is much larger than the eccentricity, so the eccentricity usually does not affect the output voltage of the sensor [1]. In order to improve the eccentricity problem, the current solution is to use the differential structure. To a certain extent, the interferences of eccentricity and other common mode parameters are eliminated by the differential circuit when the control accuracy is not high. However, the accurate control of the magnetic suspended rotor position is very important for the work state of the motor. It is necessary to study the measurement technology of magnetic suspension motors with the existing eccentricity phenomena. The structure of a single-winding magnetic suspension motor with the rotor radial position detection unit is shown in Figure 1.

Aiming at the accurate control of the rotor radial position in the magnetic suspension motor system, on the basis of the transformer model [21] and the eccentric analytical model, this paper investigates the influence of eccentricity on the ECDS by the finite element method (FEM) and experiments. Then, aiming at different structures of the experimental platform, two compensation equations are put forward, respectively. Meanwhile, for the engineering application, the orthogonal direction measured value is used as the eccentricity, and the compensation order is proposed. The experiment results show that the presented compensation method is efficient.

## 2. Mathematical Models Analyses

### 2.1. Transformer Model Analysis

The transformer model can effectively explain the coupling relationship between the probe coil of the ECDS and the conductor [21]. The probe coil constitutes inductance and resistance. The conductor surface generates eddy when the probe coil is fed with alternating current. The probe coil constitutes the primary winding of the transformer and the vortex ring can be regarded as the secondary winding [17].

$R_{1}$, $L_{1},$ and $U_{1}$ represent the coil resistance, the coil inductance, and the excitation, respectively. The eddy current generated by the probe coil on the conductor surface is considered to be a vortex ring and the influence of the vortex ring on the probe coil impedance is analyzed. $R_{2}$ and $L_{2}$ represent resistance and inductance of the conductor, where M ($M=k\sqrt{L_{1}L_{2}}$ (0 < k < 1)) is the coupling strength between the primary magnetic field and the second magnetic. The closer the distance between the probe coil and the conductor, the greater M.

Based on KVL voltage principle:

(1) $$\left\{ \begin{array}{l} R_{1}\overset{•}{I_{1}}+j\omega L_{1}\overset{•}{I_{1}}-j\omega M\overset{•}{I_{2}}=\overset{•}{U_{1}}\\ R_{2}\overset{•}{I_{2}}+j\omega L_{2}\overset{•}{I_{2}}-j\omega M\overset{•}{I_{1}}=0 \end{array}\right.$$

$L_{S}$ and $R_{S}$ represent the equivalent inductance and the equivalent resistance, which can be deduced from Equation (1):

(2) $$\left\{ \begin{array}{l} R_{S}=R_{1}+\frac{\omega^{2}M^{2}}{R_{2}^{2}+{\left(\omega L_{2}\right)}^{2}}R_{2}\\ L_{S}=L_{1}-\frac{\omega^{2}M^{2}}{R_{2}^{2}+{\left(\omega L_{2}\right)}^{2}}L_{2} \end{array}\right.$$

ω represents the angular frequency. Combining with Equation (2), with the increase of the distance between the probe coil and the conductor, $R_{S}$ slowly becomes consistent with $R_{1}$, and so does $L_{1}$. The conversion circuit in the sensor is used to convert $R_{S}$ and $L_{s}$ into an electric signal which is to determine whether the displacement changes.

### 2.2. Simplified Eccentric Analytical Model

Based on the Biot-Savart law, the magnetic induction in symmetrical axis ($B_{P}$) of a single-turn coil is a function related to the radius of the coil carrying current, the axial distance of the coil carrying current, and the excitation current.

**Figure 2:**
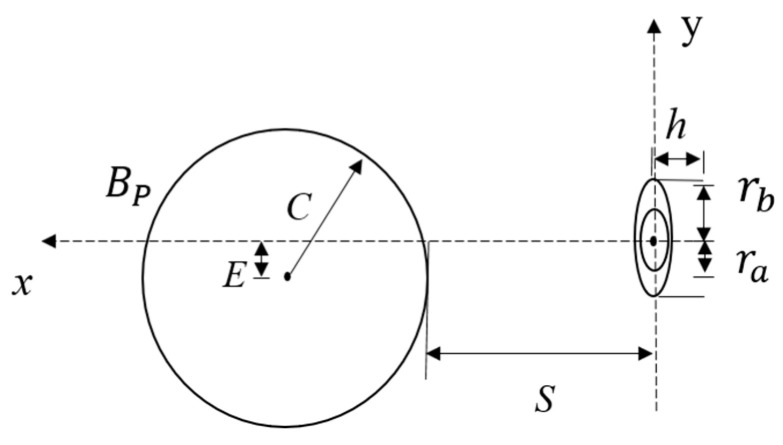
The simplified eccentric analytical model.

In order to analyze the effect of the eccentricity between the sensor and the axis of the measured cylinder on the detection, a simplified eccentric analytical model is established as shown in Figure 2. *S*, $\mu_{0}$, and *I* represent the axial distance of the coil carrying current, the permeability of vacuum, and the excitation current, respectively. *C* is the radius of the cylinder. *E* represents the eccentricity and *h* is the thickness of the coil carrying current. $r_{a}$ and $r_{b}$ represent the inner radius and the outer radius of the current-carrying coil, respectively. *N* represents the number of the coil turns.

The current through a unit on the section of the probe coil is

(3) $$i=\frac{NI}{\left(r_{b}-r_{a}\right)h}dxdy$$

The magnetic induction intensity generated by this unit can be obtained by the Biot-Savart law.

(4) $$dB_{P}=\frac{\mu_{0}x^{2}}{2{\left(x^{2}+y^{2}\right)}^{3/2}}\frac{NI}{\left(r_{b}-r_{a}\right)h}dxdy$$

Introducing the effect of *E*, the magnetic field generated by the probe coils with *N* turns is formed by the superposition of the magnetic field generated by the unit current.

(5) $$B_{P}=\int dB_{P}=\frac{\mu_{0}NI}{2\left(r_{a}-r_{b}\right)h}\left\{\left(S+h+C-\sqrt{C^{2}-E^{2}}\right)\ln\left|\frac{r_{b}+\sqrt{r_{b}^{2}+h}}{r_{a}+\sqrt{r_{a}^{2}+h}}\right|-\left(S+C-\sqrt{C^{2}-E^{2}}\right)\ln\left|\frac{r_{b}+\sqrt{r_{b}^{2}+\left(S+C-\sqrt{C^{2}-E^{2}}\right)}}{r_{a}+\sqrt{r_{a}^{2}+\left(S+C-\sqrt{C^{2}-E^{2}}\right)}}\right|\right\}$$

Combining with Equation (5), $B_{P}$ is correlated with *N*, *I*, *S*, $r_{a}$, $r_{b}$, *h*, *C*, and *E*. In order to analyze the effect of *E* and *C* on the detection, the $B_{P}$-S curve is obtained by keeping other parameters constant. Normalized $B_{P}$ and normalized *S* are defined as:

(6) $$\left\{ \begin{array}{l} B_{P}=\frac{B_{Pi}-B_{P\min}}{B_{P\max}-B_{P\min}}\\ S=\frac{S_{i}-S_{\min}}{S_{\max}-S_{\min}} \end{array}\right.$$

**Figure 3:**
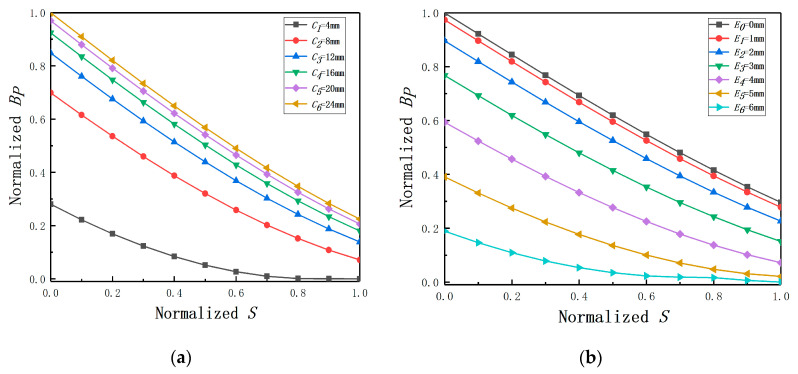
The $B_{P}$-*S* curves under condition of: (**a**) different radii; (**b**) different eccentricities.

**Figure 4:**
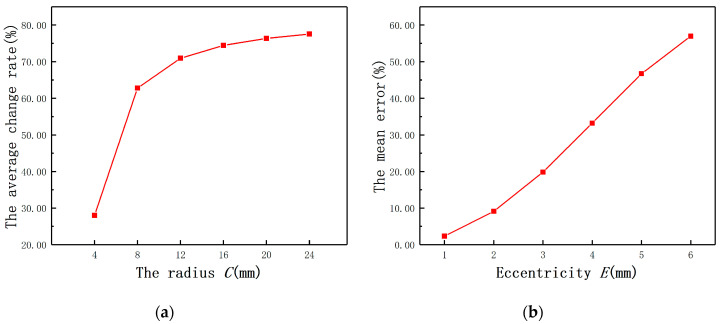
(**a**) The average change rates of $B_{P}$ under different radii; (**b**) the mean errors of $B_{P}$ under different eccentricities.

In Figure 3a, *C* is set as 4, 8, 12, 16, 20, 24 mm under fixed *E* (*E* = 2 mm). $r_{a}$ is 3 mm, $r_{b}$ is 4 mm, and *h* is 3 mm. The $B_{P}$-*S* curves of different radii cylinders are not coincident with each other under fixed *E* as shown in Figure 4a. Combining with Equation (2) and the eccentric analytical model, different *C* can cause different *M*, and this finally affects $L_{S}$ and $R_{S}$. Another conclusion can be drawn that with the increase of *C*, the effect of *E* tends to be smaller. The average change rate is defined as $B_{P\left(S=0\right)}-B_{P\left(S=1\right)}$. As shown in Figure 4a, the effect of *E* tends to be smaller with the increase of *C* and the average change rate of $B_{P}$ remains approximately constant when the *C*-$r_{b}$ ratio is over 3, which provides the basis for the type selection of the ECDS.

In Figure 3b, *E* is set as 0, 1, 2, 3, 4, 5, 6 mm under fixed *C* (*C* = 30 mm) and the curves of different *E* are inconsistent under fixed *C*. A similar conclusion can also be drawn that with the decrease of *E*, the effect of *E* on displacement measurement becomes weaker. In order to make the quantitative analysis, the curve of *E* = 0 mm is used as a standard curve and the mean error is defined as the average of $B_{P\left(E\neq 0mm\right)}-B_{P\left(E=0mm\right)}$. As shown in the Figure 4b, the mean error of $B_{P}$ (*E* = 3 mm) is one order of magnitude bigger than the mean error of $B_{P}$ (*E* = 1 mm), which means *E* increasingly impacts on the displacement measurement when the *E*-*C* ratio is over 0.1.

Based on the conclusions of the simplified eccentric analytical model, the radius of the measured cylinder and the size of the eccentricity have influences on the magnetic induction in the symmetrical axis of the probe coil and subsequently influence the eddy current distribution on the conductor. For a magnetic suspension motor system, the rotor eccentricity under a fixed radius is the main cause of measurement error.

## 3. Simulation Analyses

**Figure 5:**
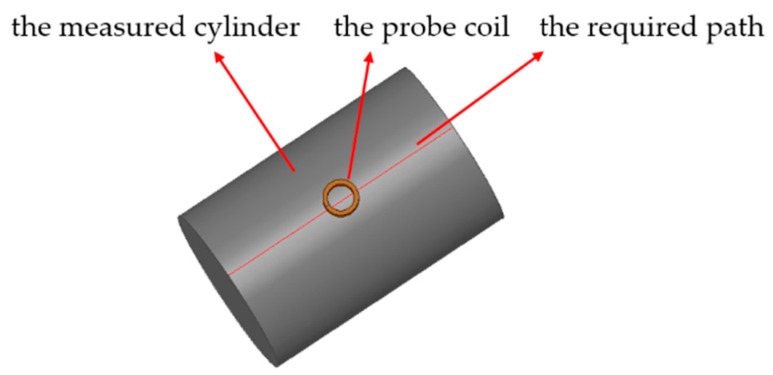
3-D finite element analysis model.

The influences of the cylinder radius and the eccentricity on detection was investigated by the FEM, ANSYS Maxwell. The solution type Eddy Current was selected. The material of the coil and the cylinder were defined as copper and aluminum. The inner radius, outer radius, and thickness of the probe coil are 3, 4, and 3 mm, respectively. The length of the cylinder is 60 mm. The excitation frequency is 10 kHz, and the solution region is 300%. Considering the effect of skin on the eddy current distribution, the mesh operation for the measured cylinder was defined as skin depth subdivision. In the solution of the eddy current field, the information of the eddy density distribution, magnetic inductive intensity distribution, coil inductance, and other information in the postprocessing results were mainly extracted. Before the solution, the required path was set in advance as shown in Figure 5.

**Figure 6:**
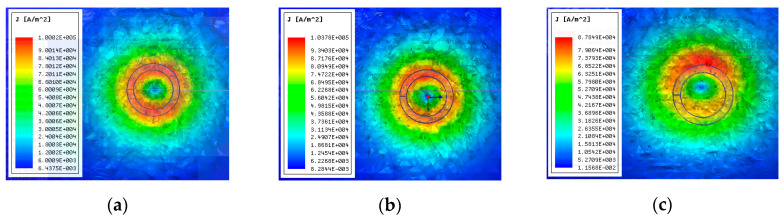
The eddy current distributions under different eccentricities: (**a**) *E* = 0 mm; (**b**) *E* = 1 mm; (**c**) *E* = 5 mm.

**Figure 7:**
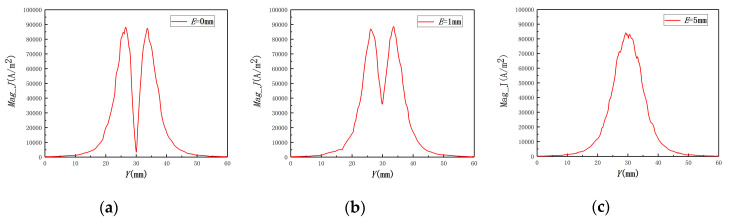
The distribution curves of the eddy current density under different eccentricities: (**a**) *E* = 0 mm; (**b**) *E* = 1 mm; (**c**) *E* = 5 mm.

To validate the effect of *E* on the induced eddy current, the eddy current distributions under different *E* were carried out by the FEM, as shown in Figure 6. The distribution curves of the eddy current density on the required path are shown in Figure 7.

As shown in Figure 6 and Figure 7, the eddy current distribution and amplitude are different when the eccentricity changes. Based on Faraday electromagnetic induction law, the secondary magnetic field caused by the eddy current will affect the variety of the primary magnetic field, while *E* and *C* affect the secondary magnetic field. This will finally affect $L_{s}$ and the electric signal output.

### 3.1. Change Laws of Coil Equivalent Impedance under Different Eccentricities and Lift-Off

In order to investigate the effect of *E* on the detection, it also could be transformed into the study of the change law of the coil equivalent impedance under different *E* and lift-off. *E* was set as 0, 1, 2, 3, 4, 5 mm, respectively, and the radius of the measured cylinder was 30 mm. The lift-off refers to the distance moved along the axis of the probe coil. The lift-off was set as 1, 2, 3, 4, and 5 mm, respectively.

**Figure 8:**
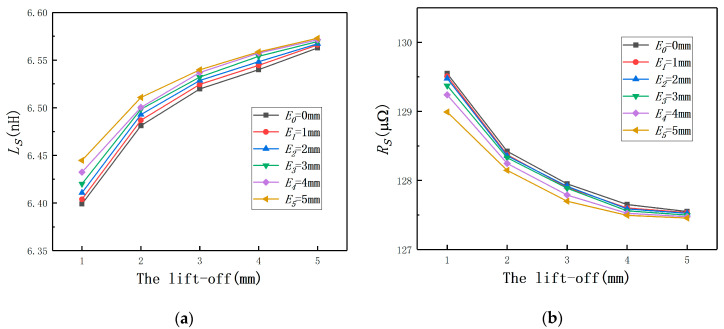
The change laws of the coil equivalent impedance under different eccentricities: (**a**) the coil equivalent inductance; (**b**) the coil equivalent resistance.

By using Maxwell, it could be found that $L_{s}$ increases with the lift-off until to the probe coil self-inductance, as shown in Figure 8a. The variation of $R_{s}$ is contrary to $L_{s}$ and also remains unchanged at the end as shown in Figure 8b. The results of *E* = 0 mm proved the validity of the transformer model. In addition, the curves of different *E* prove that no effects are found on the applicability and effectiveness of the transformer model.

**Figure 9:**
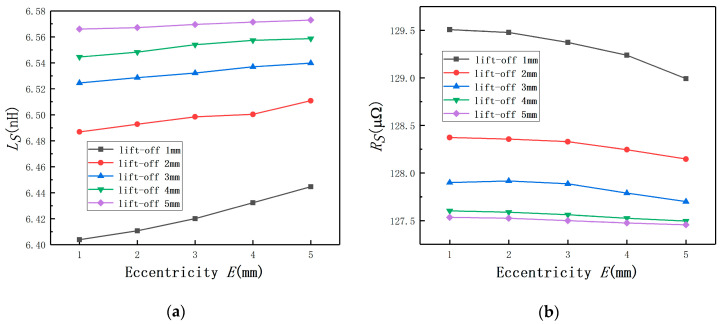
The change laws of the coil equivalent impedance under different lift-off: (**a**) coil equivalent inductance; (**b**) coil equivalent resistance.

When the lift-off remains constant, the change laws of $L_{s}$ are shown in Figure 9a. It can be seen that, with the increase of *E*, $L_{s}$ increases. It means that the effect of *E* on the eddy current sensor is the same with that of the lift-off. As the coil equivalent impedance contains two components, the variation laws of $R_{s}$ are also shown in Figure 9b. It also can be concluded that *L* and the lift-off have the same influence tendency for the eddy current displacement measurement. It means *E* will lead to a bigger measured value.

**Figure 10:**
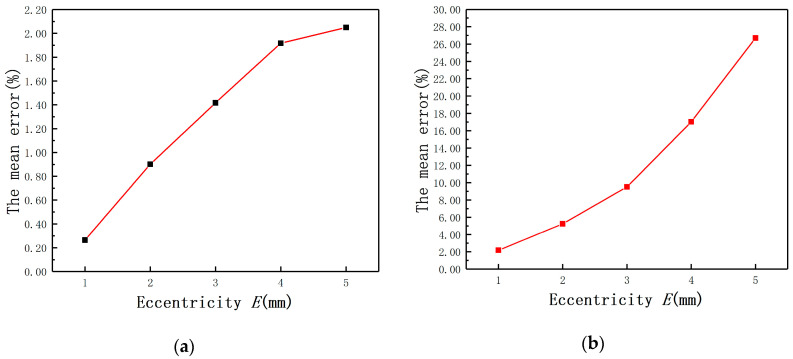
The mean errors with different eccentricities: (**a**) $L_{s}$; (**b**) $R_{s}$.

In order to undertake the quantitative analysis, the effect of *E* on the displacement measurement was compared by the mean error. The mean error of $L_{s}$ under different *E* was defined as the average of $L_{S\left(E\neq 0\mathrm{mm}\right)}-L_{S\left(E=0\mathrm{mm}\right)}$ and the mean error of $R_{s}$ under different *E* was defined as the average of $R_{S\left(E\neq 0\mathrm{mm}\right)}-R_{S\left(E=0\mathrm{mm}\right)}$. As shown in Figure 10, the mean error of $L_{s}$ (*E* = 4 mm) is one order of magnitude bigger than the mean error of $L_{s}$ (*E* = 1 mm), so is $R_{s}$. It means that the measurement error must be compensated when the *E*-*C* ratio is over 0.1. The conclusion is consistent with the result of the mathematical model analysis.

**Figure 11:**
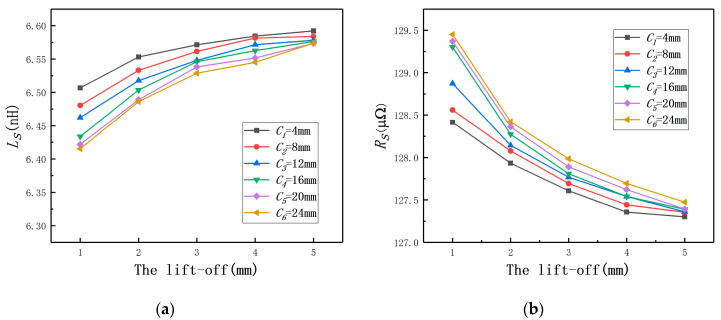
The change laws of the coil equivalent impedance under different radii: (**a**) the coil equivalent inductance; (**b**) the coil equivalent resistance.

**Figure 12:**
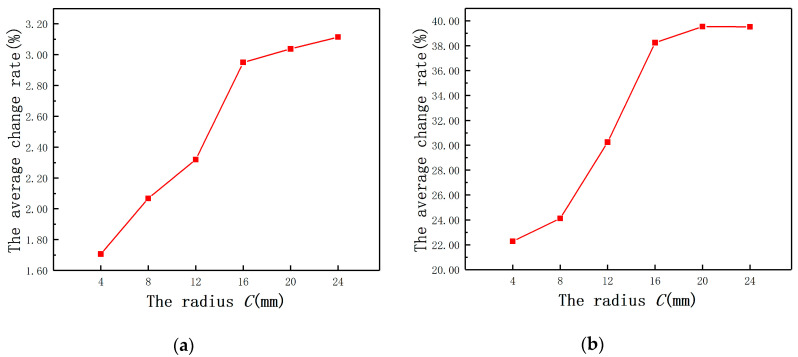
The average change rates under different radii: (**a**) $L_{s}$; (**b**) $R_{s}$.

To verify the reliability of the type selection of the ECDS in the analysis of mathematical model, *C* was set as 4, 8, 12, 16, 20, and 24 mm, respectively, and the probe radius was 4 mm. By using Maxwell, it could be found that $L_{s}$ increases with *C* until to the probe coil self-inductance, as shown in Figure 11a. The variation of $R_{s}$ is contrary to $L_{s}$ and $R_{s}$ also remains unchanged at the end as shown in the Figure 11b. As shown in Figure 12, when the *C*-$r_{b}$ ratio is over 3, the average change rate of $L_{s}$ remains approximately constant, so does $R_{s}$. The result is consistent with the conclusion in the previous quantitative analysis.

### 3.2. Change Laws of Impedance Plane under Different Excitation Frequencies

As the previous section suggests, *E* will cause a bigger measured value. The impedance plane method is an effective way for presenting results in the eddy current testing [22] and can be considered as another perspective to discuss the effect of *E* on the eddy current displacement measurement. The normalized *R* and *X* are defined using Equation (7):

(7) $$\left\{ \begin{array}{l} R=\frac{R_{S}-R_{0}}{X_{0}}\\ X=\frac{X_{S}}{X_{0}} \end{array}\right.$$

$X_{S}$ ($X_{S}$ = $\omega L_{S}$) is the coil equivalent reactance and $R_{S}$ is the coil equivalent resistance. The coil self-reactance is expressed by $X_{0}$ ($X_{0}=\omega L_{0}=2\pi fL_{0}$). $L_{0}$ and $R_{0}$ are the coil self-resistance and self-inductance. ω and f represent the angular frequency and the excitation frequency, respectively. Finally, the impedance plane is formed by drawing normalized *R* vs. normalized *X*.

**Figure 13:**
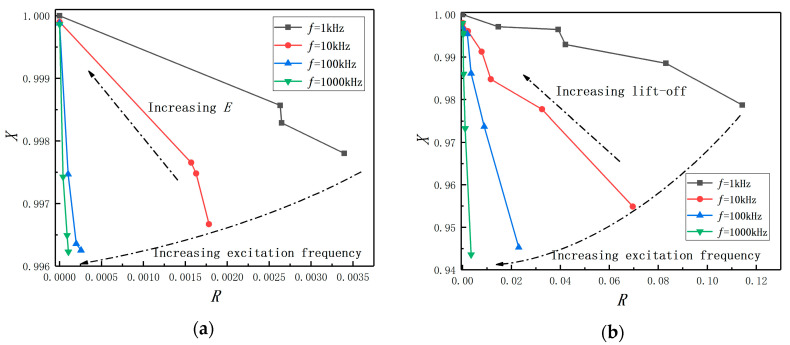
The normalized impedance plane under condition of (**a**) varying eccentricity; (**b**) varying lift-off.

In Figure 13a, *E* is set as 0, 1, 3, and 5 mm, respectively, under fixed lift-off (2 mm). The lift-off is set as 0,1, 2, 3, 4, and 5 mm, respectively, under fixed *E* (*E* = 0 mm), as shown in Figure 13b. Combining with Equation (7), the coil normalized impedance will be located on the point (0, 1) as a reference point. From the normalized impedance plan graphs, the coil normalized impedance gets closer to the reference point with the increase of *E* under different f. Analogously, the normalized impedance gets closer to the reference point with the increase of the lift-off under different f, as shown in Figure 13b. Thus, the change law of the coil impedance with *E* is similar to the change law of the coil impedance with lift-off. Therefore, it can be concluded that the effect of *E* on the probe coil equivalent impedance is similar to the effect of the lift-off, which means that *E* will cause a bigger electrical signal in the eddy current displacement measurement. The conclusion is consistent with the conclusion of the previous simulation analysis.

## 4. Experimental Process and Results

### 4.1. Eccentric Experimental Platform

**Figure 14:**
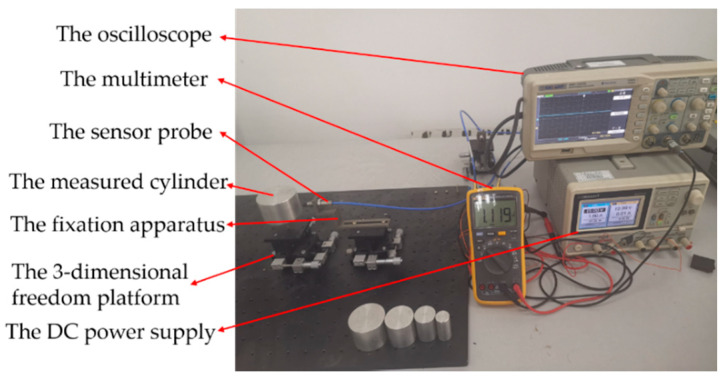
The eccentric experimental platform.

In the eccentric experiment, the experimental platform not only needs to realize the change of moving distance, but also the change of *E*. Thus, a type of 3-dimensional freedom platform was selected. The measured cylinder was placed on one of the 3-dimensional freedom platforms, and the eddy current displacement sensor was placed on another one. Their center axes intersected at right angles. The K9000XL with the range of 2 mm was selected as the ECDS, the LY12 aluminum alloy cylinder was selected as the measured cylinder and the location precision of the freedom platform was 0.01 mm. The radius of the probe coil was 4 mm. The probe of the ECDS was fixed on the fixation apparatus through the threaded connection. The moving distance was set through the movement of *X* axis of the 3-dimensional freedom platform with fixed the measured cylinder and *E* was set through the movement of *Y* axis of another freedom platform, as shown in Figure 14. The moving distance and the lift-off have the same physical meaning.

### 4.2. Relationship between Output and Moving Distance under Different Eccentricities

**Figure 15:**
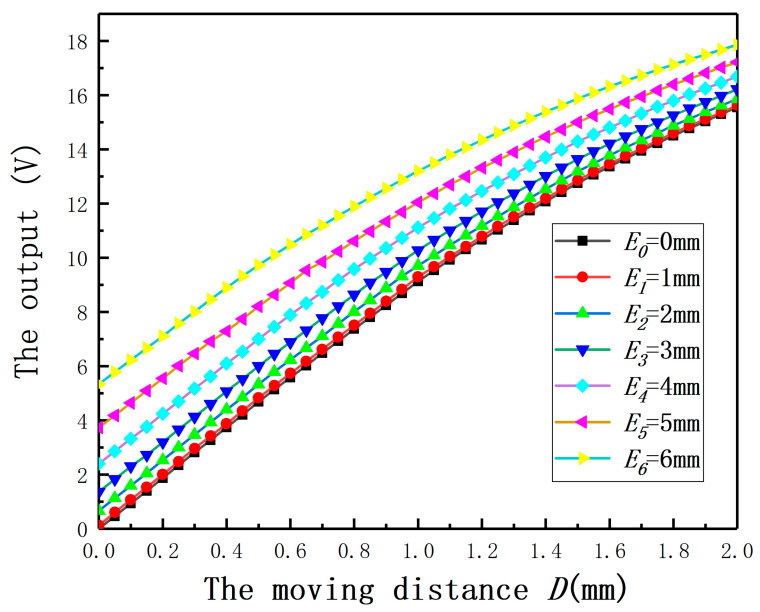
The relationship between the output and the moving distance under different eccentricities.

In order to validate the effect of *E* on the displacement measurement of the ECDS, *E* was set as 0, 1, 2, 3, 4, 5, 6 mm and the radius of the measured cylinder was 30 mm. To the specific material and measured cylindrical specimens with different radii, a calibration experiment is needed to get the output characteristic curve for the ECDS firstly. Thus, the experiment (*E* = 0 mm) was used as the control experiment. The output curves under different *E* are shown in Figure 15. If *E* has no effect on the measurement, the output curves under different *E* should be coincident with the output characteristic curve. According to the results, the output curves are above the output characteristic curve. It means *E* leads to a bigger measured value. Comparing with the results of the experiments and the simulation, the conclusion about the influence of *E* on the measured value is verified. Additionally, the measurement error will increase with the increase of *E*.

**Figure 16:**
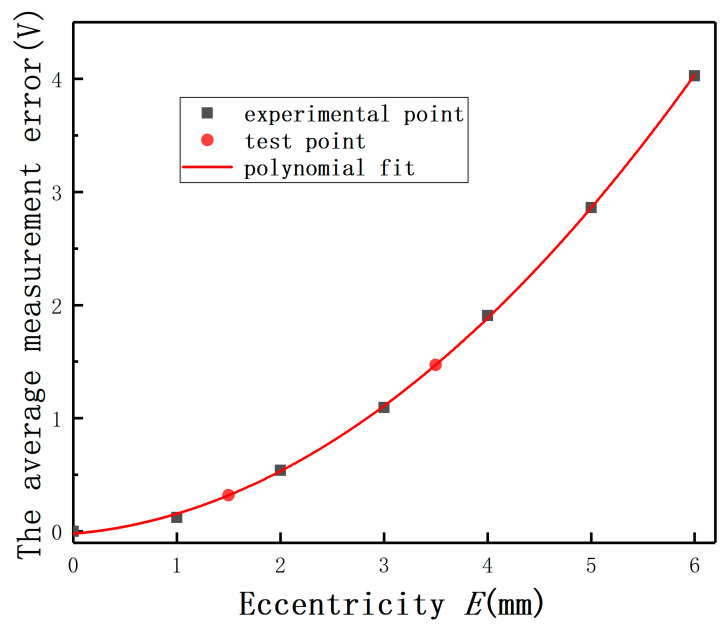
The average measurement errors with different eccentricities.

In order to undertake the quantitative analysis and reduce the difficulty of the measurement error analysis, the effect of *E* on the displacement measurement was compared by the average measurement error. The average measurement error of the output under different *E* is defined as the average of ${\mathrm{the}\;\mathrm{output}}_{\left(E\neq 0\mathrm{mm}\right)}-{\mathrm{the}\;\mathrm{output}}_{\left(E=0\mathrm{mm}\right)}$. As shown in Figure 16, the average measurement error of the output (*E* = 3 mm) is one order of magnitude bigger than the average error (*E* = 1 mm). It means that the measurement error must be compensated when the *E*-*C* ratio is over 0.1. This conclusion is consistent with the results of the mathematical model analysis and the simulation analysis.

With polynomial fit method, the change law of the average measurement error with *E* is shown in Figure 16.

(8) $$y=a+bx+cx^{2}$$

Among that, a=−0.0198, b=0.0761, c=0.0999. The variable *x* represents *E* and the variable *y* represents the average measurement error. The residual sum of squares is 2.51 × 10^−3^, and the coefficient of determination is 0.999. The results show that the change law of the average measurement error with *E* can be described with the quadratic polynomial, as shown in Figure 16. Two test points were used to verify the correctness of the conclusion.

**Figure 17:**
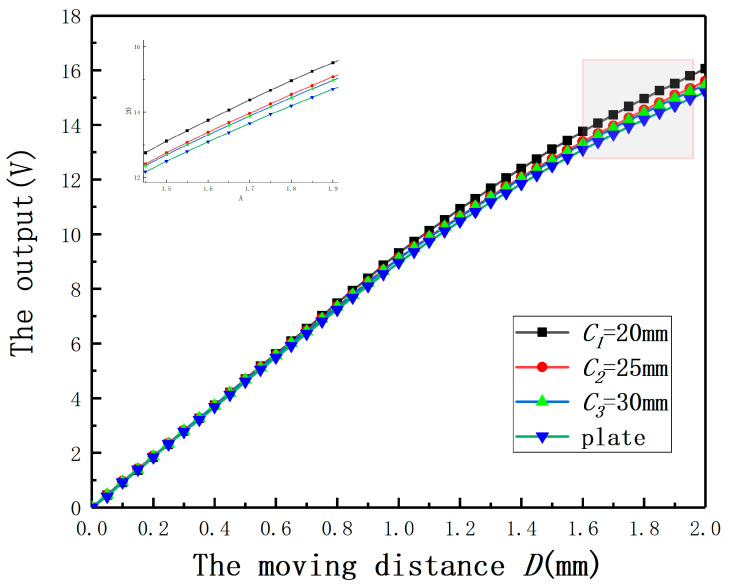
The relationship between the output and the moving distance under different radii.

To validate the analysis results of the eccentric analytical model and the simulation about the effect of *C* on displacement measurement, firstly, the output curves under *E* = 0 mm with different radii were obtained as shown in Figure 17. The radius of the measured plate can be considered as infinite. The following conclusions can be drawn: (1) when the radius of the measured cylinder changes, the output curve must be recalibrated. (2) The output curve is getting closer to the output curve of the plate with the increase of the measured cylinder radius.

**Figure 18:**
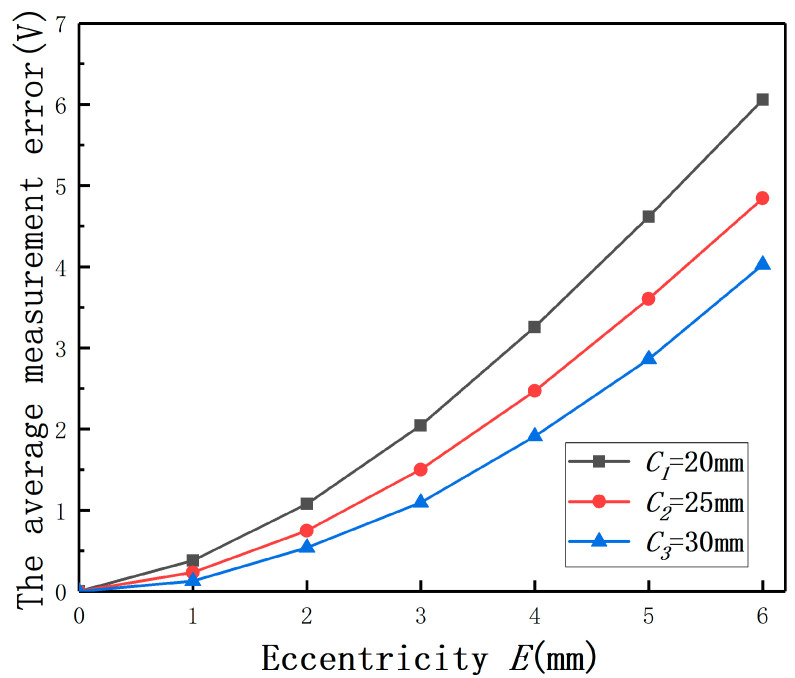
The average measurement errors under different radii.

Then, the curves between the average measurement error and *E* under different radii are shown in Figure 18. The curves once again prove that there are good quadratic polynomial relations between average the measurement error and *E*. The measurement errors by *E* becomes smaller with the increase of the measured cylinder radius. As shown in Figure 18, the average measurement error is one order of magnitude bigger than the average measurement error (*E* = 1 mm) when the *E*-*C* ratio is over 0.1. Through changing the measured cylinder radius, the previous conclusion about the *E*-*C* ratio is verified.

### 4.3. Compensation Method for Measurement Errors

Drawing on the experimental results, *E* will produce the measurement errors that increase with the increase of *E*. Thus, it is necessary to compensate for the measurement errors. Due to the measurement error, ΔU is the function of the moving distance (*D*) and *E*, the compensation equation is obtained through using the following steps.

Firstly, through the principle of least square method, the modified equation about ΔU and *D* is proposed. With the range of *E* is from 1 to 6 mm, $\frac{d\Delta U}{dD}$ is −0.0334, −0.1939, −0.3818, −0.7025, −1.1472, and −1.6867, respectively.

(9) $$\left\{ \begin{array}{l} \Delta U_{1}=-0.0334D+0.1563\\ \Delta U_{2}=-0.1939D+0.7347\\ \Delta U_{3}=-0.3818D+1.4765\\ \Delta U_{4}=-0.7025D+2.6115\\ \Delta U_{5}=-1.1472D+4.0094\\ \Delta U_{6}=-1.6867D+5.7133 \end{array}\right.$$

Considering the effect of *E*, *E* is introduced into the compensation equation.

(10) $$\frac{d\Delta U}{dD}=-0.0522E^{2}+0.0382E+0.0332$$

Integrating with respect to *D*, Equation (9) can be obtained.

(11) $$\Delta U=-0.0522DE^{2}+0.0382DE-0.0332D+C\left(E\right)$$

Then, based on polynomial fitting and Equation (7), the concrete expression of C(E) can be obtained.

(12) $$C\left(E\right)=0.1472E^{2}+0.0765E-0.0498$$

Thus, the compensation equation is:

(13) $$\Delta U=-0.0522DE^{2}+0.0382DE-0.0332D+0.1472E^{2}+0.0765E-0.0498$$

Last, the final compensation equation is obtained after verification calculation and adjustment for individual parameters.

(14) $$\Delta U=-0.0522D+0.0382\frac{D}{E}-0.0332\frac{D}{E^{2}}+0.1472+\frac{0.0765}{E}-\frac{0.0498}{E^{2}}$$

**Figure 19:**
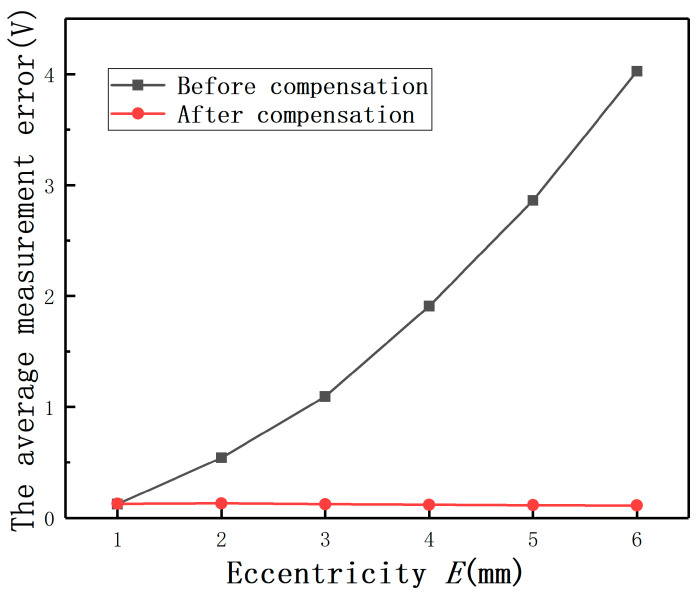
The average measurement errors before and after compensation.

**Figure 20:**
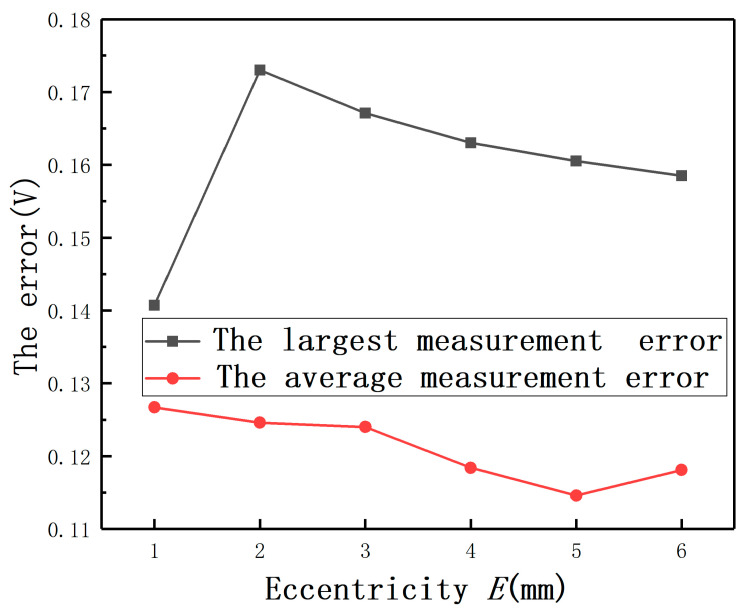
The largest measurement errors and the average measurement errors after compensation.

**Figure 21:**
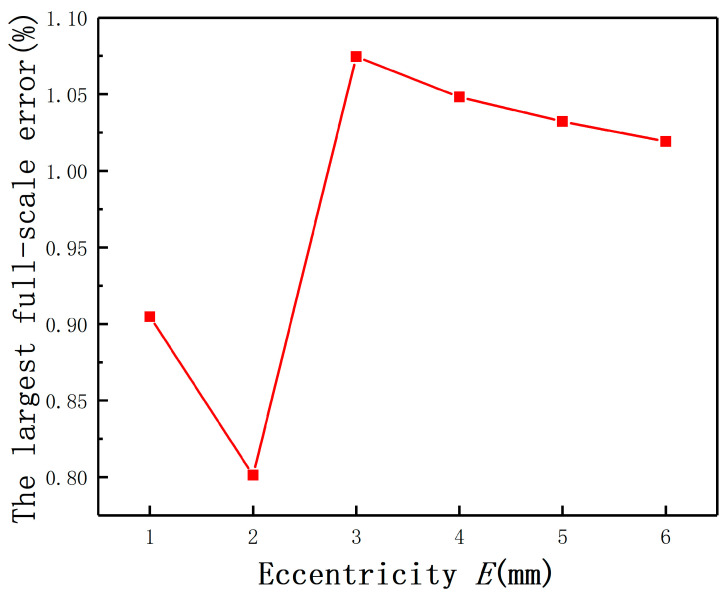
The largest full-scale errors after compensation.

The average measurement errors under different *E* with compensation and without compensation are shown in Figure 19**.** If the largest measurement error is defined as the maximum value of $the\;output_{\left(E\neq 0\mathrm{mm}\right)}-the\;output_{\left(E=0\mathrm{mm}\right)}$, the largest full-scale error is defined as the largest measurement error/$\left(the\;output_{\left(D=2mm,E=0mm\right)}-the\;output_{\left(D=0mm,E=0\mathrm{mm}\right)}\right)$. The largest measurement errors after compensation under different *E* are shown as Figure 20. It can be found that the largest full-scale error under *E* = 2 mm is less than 0.8%, as shown in Figure 21. Thus, the results show that this method has the advantage of reducing the measurement errors greatly.

**Figure 22:**
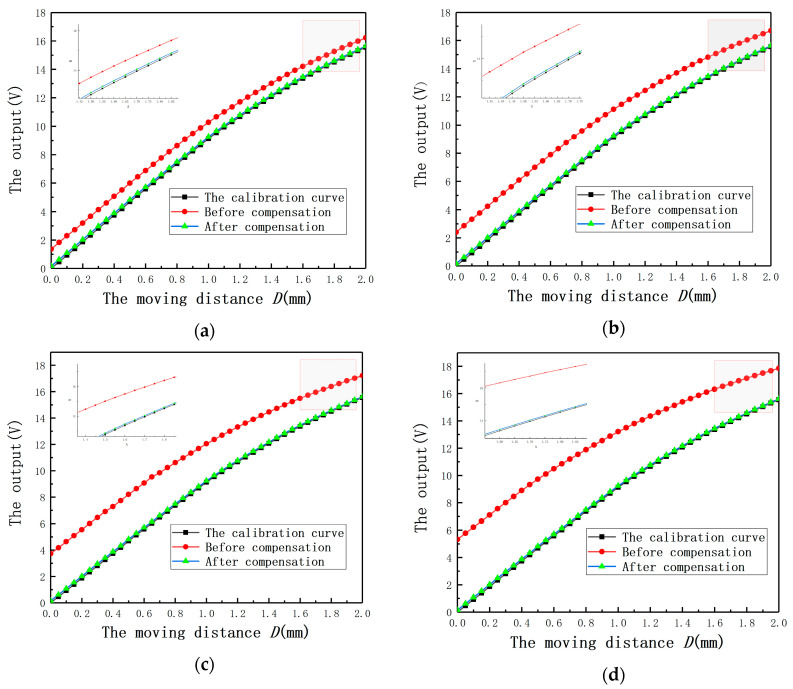
The output curves with and without compensation under different eccentricities: (**a**) *E* = 3 mm; (**b**) *E* = 4 mm; (**c**) *E* = 5 mm; (**d**) *E* = 6 mm.

In order to show the compensation effect more intuitively, the output curves under different *E* before and after compensation are shown in Figure 22. It can be found that the largest average measurement error under different *E* is less than 0.18 V. The results again prove that Equation (14) is effective to compensate the measurement errors under *E*.

### 4.4. Compensation for Measurement Errors in Differential Structure

**Figure 23:**
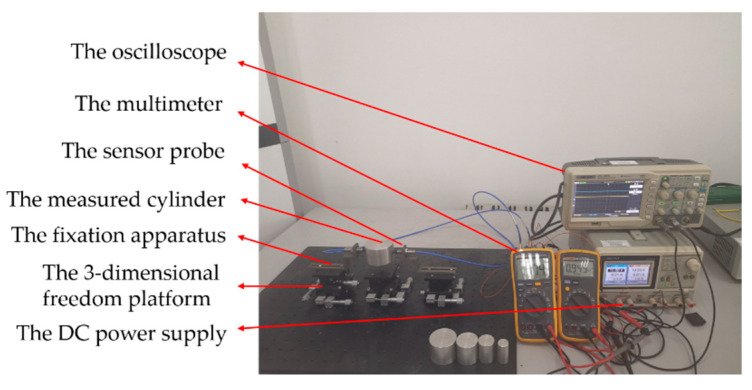
The differential structure eccentric experimental platform.

The relationship between the output and the moving distance is not perfectly linear as shown in Figure 15. To improve the system linearity and obtain better compensation results, the differential structure was adapted. The system had two sensors and they were separated by 180 degrees (opposite each other) as shown in Figure 23. The range of the sensors was changed from 0–2 to −1–1 mm by adjusting zero potentiometer. The measured cylinder (*C* = 30 mm) was fixed at the position where the output voltages of both sensors were zero. Then the average of the difference between these two sensors was the final output.

**Figure 24:**
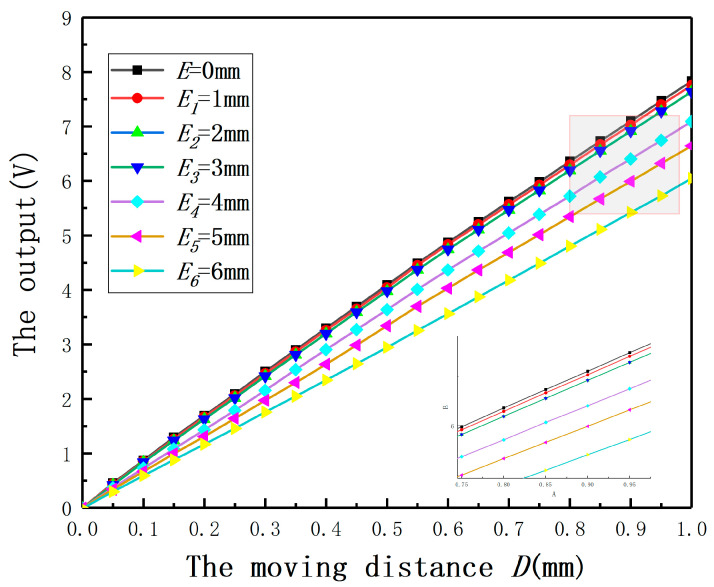
The relationship between the output and the moving distance under different eccentricities.

**Figure 25:**
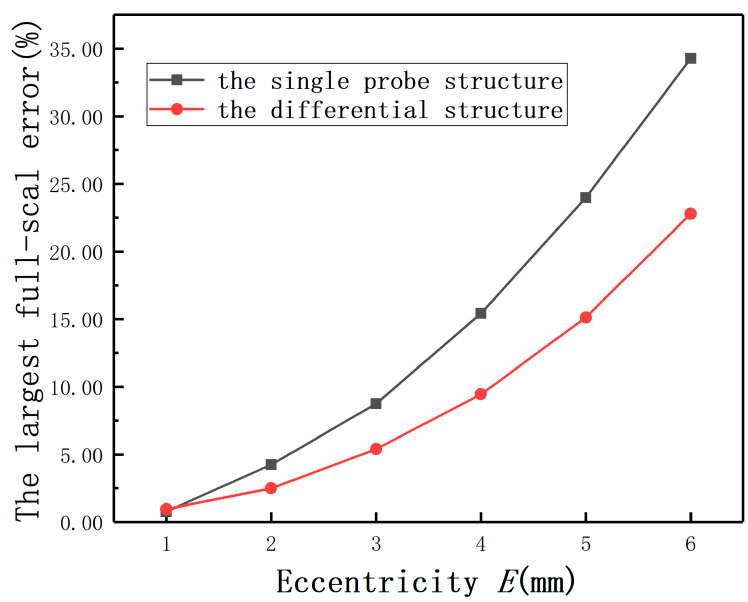
The largest full-scale errors with different eccentricities.

*E* was set as 0, 1, 2, 3, 4, 5, and 6 mm, respectively. The results are shown in Figure 24. The experiment (*E* = 0 mm) was used as the control experiment. Due to the nonlinearity of the sensor’s output curve, the output curves of the differential structure under different *E* are below the output characteristic curve. Comparing with Figure 15, the linearity of the system is significantly improved by using the differential structure. As shown in Figure 25, the largest full-scale error is reduced by more than 40% under *E* = 2 mm after employing the differential structure.

It is still necessary to compensate for the measurement errors under different *E*. The compensation method in the differential structure is the same as the method of Section 4.3. The compensation equation is:

(15) $$\Delta U=0.0628D-0.11\frac{D}{E}+0.1281\frac{D}{E^{2}}+\frac{0.0131}{E}-\frac{0.0093}{E^{2}}+0.003$$

**Figure 26:**
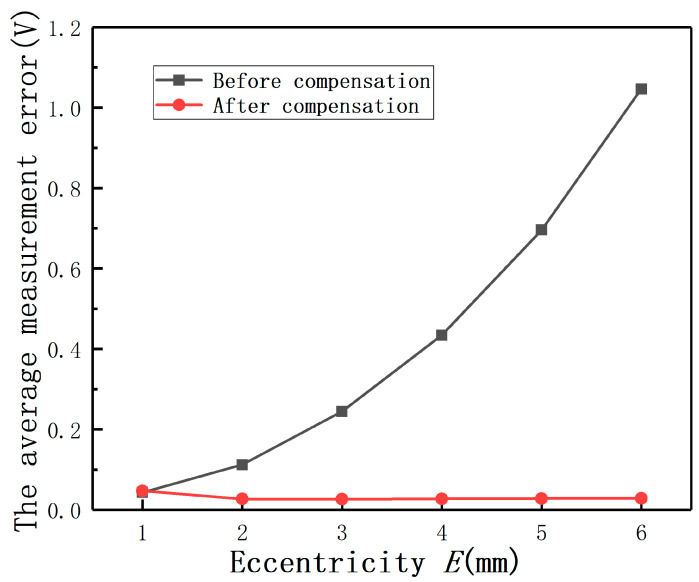
The average measurement errors before and after compensation.

**Figure 27:**
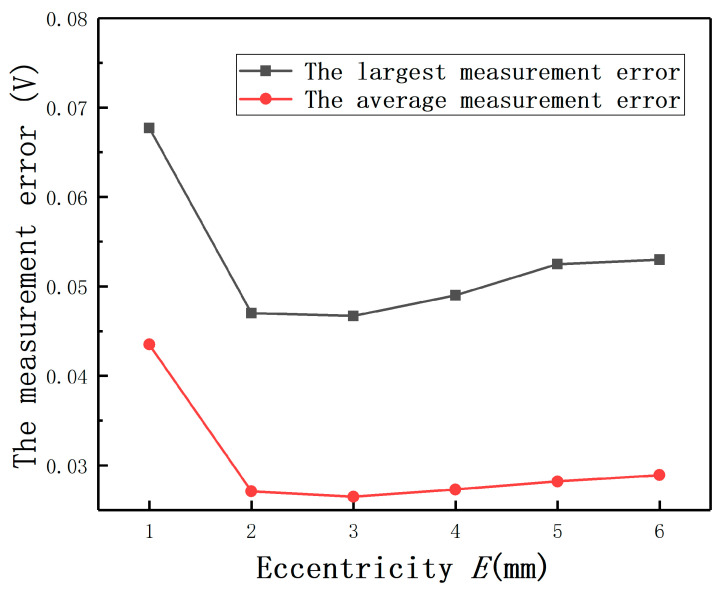
The largest measurement errors and the average measurement errors after compensation.

**Figure 28:**
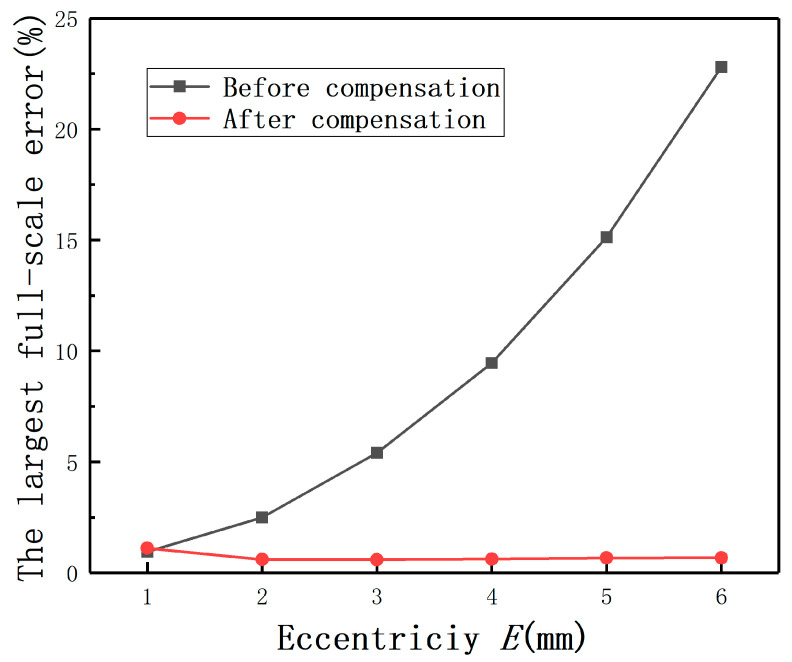
The largest full-scale errors before and after compensation.

The average measurement error under different *E* with compensation and without compensation are shown in Figure 26. The largest measurement errors after compensation under different *E* are shown in Figure 27. It also can be found that the largest full-scale error under *E* = 2 mm is less than 0.6%, as shown in Figure 28. Thus, the results show that this method is also effective for the differential structure.

**Figure 29:**
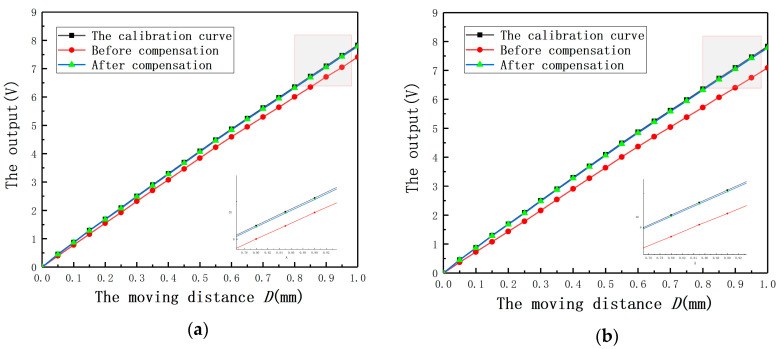

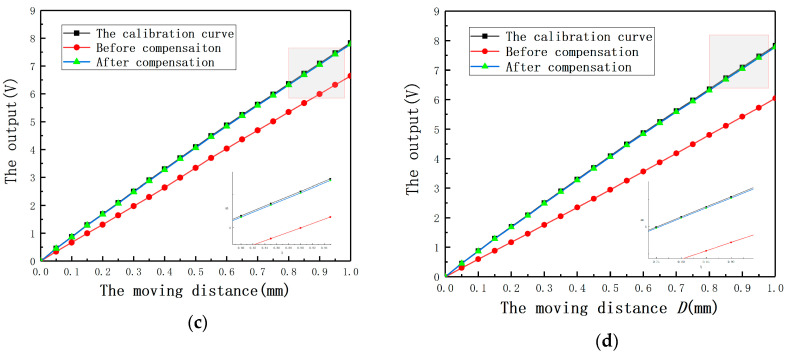
The output curves before and after compensation under different eccentricities: (**a**) *E* = 3 mm; (**b**) *E* = 4 mm; (**c**) *E* = 5 mm; (**d**) *E* = 6 mm.

In order to show the compensation effect more intuitively, the output curves under different *E* with and without compensation are shown as Figure 29. It can be obtained that the largest measurement errors under different *E* are less than 0.09 V. The results again prove that Equation (15) is effective to compensate for the measurement errors under *E*.

## 5. Application of Compensation Method in Magnetic Suspension Motor

**Figure 30:**
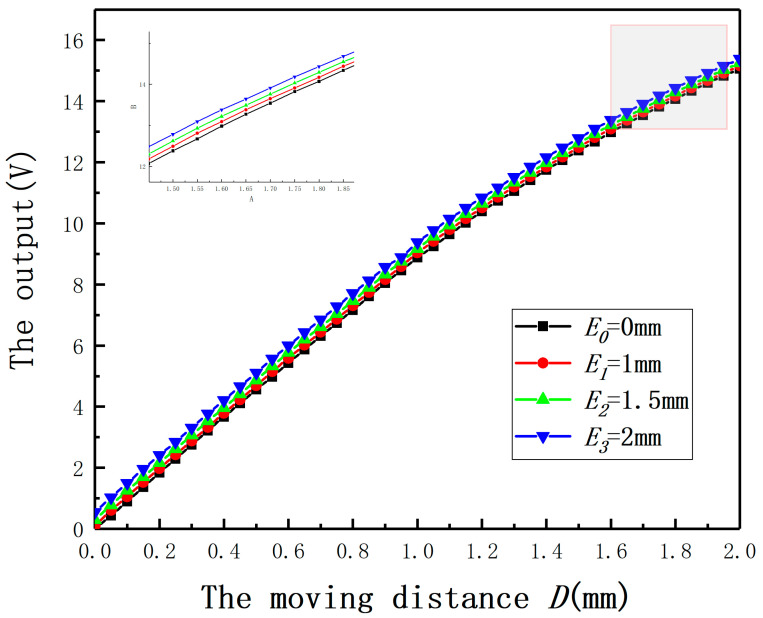
The relationship between the output and the moving distance under different eccentricities.

**Figure 31:**
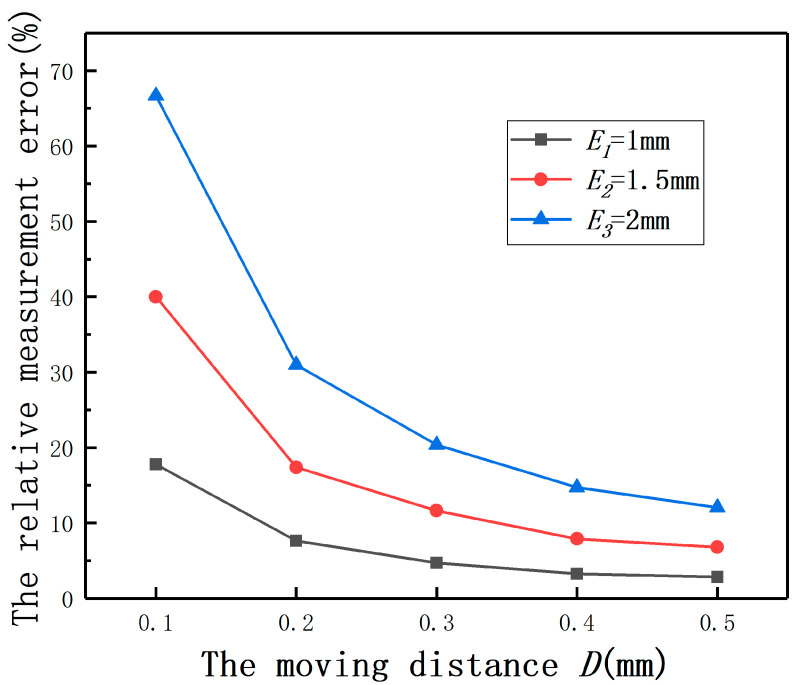
The relative measurement errors under different eccentricities.

The length of air gap has a great influence on the performance and reliability of a magnetic suspension motor. If the air gap is too large, the magnetic resistance will increase. The excitation current required to achieve the same magnetic field strength will increase greatly, and the excitation loss will also increase greatly. The power factor of the motor will decrease significantly, and the performance of the motor will deteriorate. In order to reduce the excitation current and improve the power factor, the air gap should be minimized. Generally, the air gap of a magnetic suspension motor is about 2 mm and the radial position control accuracy is 5‰. However, if the rotor radius is 30 mm, when the air gap is within 2 mm, the hardware compensation accuracy of the differential structure is 2.49%, which is difficult to meet the control accuracy, as shown in Figure 25. To further illustrate why *E* cannot be ignored in the displacement measurement of magnetic suspension motor, the effect of *E* on the displacement measurement is described by the relative measurement error. The relative measurement error is defined as $({\mathrm{the}\;\mathrm{output}}_{\left(E\neq 0\mathrm{mm}\right)}-{\mathrm{the}\;\mathrm{output}}_{\left(E=0\mathrm{mm}\right)})/{\mathrm{the}\;\mathrm{output}}_{\left(E=0\mathrm{mm}\right)}$. As shown in Figure 30 and Figure 31, it can be found that the relative measurement error cannot be ignored.

Equation (14) is mainly for large eccentricity, so a compensation equation for small eccentricity is needed. The radius of measured cylinder was 30 mm. *E* was set as 1, 1.5, and 2 mm, respectively. The compensation method for small eccentricity is the same as the method of Section 4.3. The compensation equation is:

(16) $$\Delta U=-0.00055\times \frac{D}{E}+0.003\times D$$

**Figure 32:**
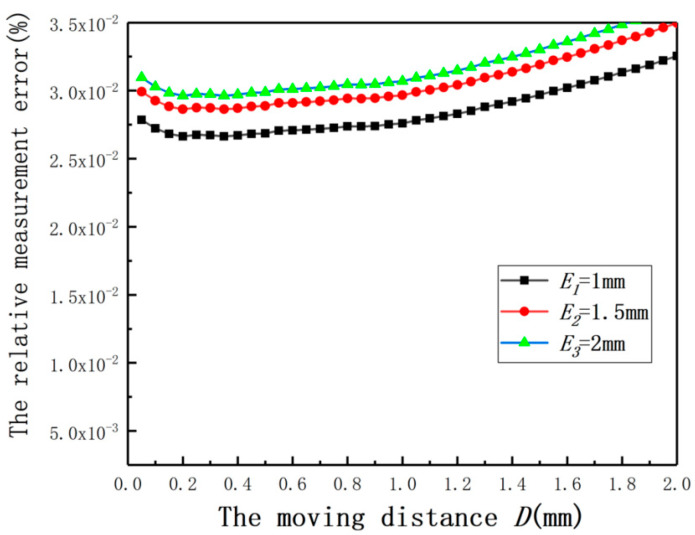
The relative measurement errors after compensation under different eccentricities.

In order to validate the effectiveness of Equation (16), the relative measurement errors under different *E* after compensation are shown in Figure 32. The results prove that Equation (16) is effective to compensate for the measurement errors under small eccentricity.

**Figure 33:**
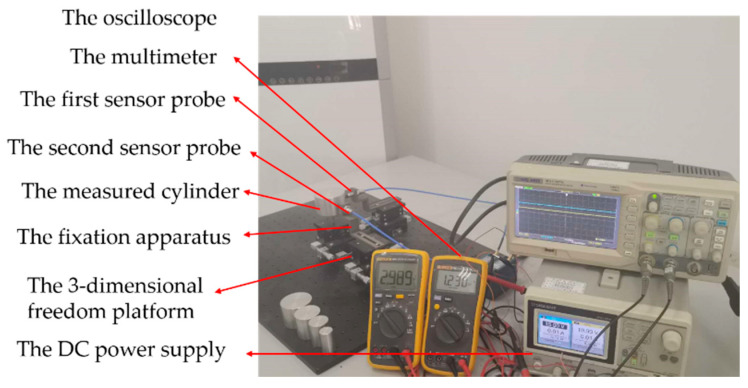
The orthogonal structure eccentric experimental platform.

The paper puts forward the compensation method to realize the eccentricity compensation, but the compensation method is based on a known eccentricity. Obviously, the compensation equation cannot be directly applied to the displacement measurement of the magnetic suspension motor system. To obtain the value of eccentricity, as shown in Figure 33, the system had two sensors and they were separated by 90 degrees. The sensors arranged in *X* and *Y* directions were used to detect the radial displacement in both directions, respectively, and the measured value of each other was used as the eccentricity of the other side to compensate the measurement errors. In theory, the effect of measurement error caused by eccentricity can be reduced.

**Figure 34:**
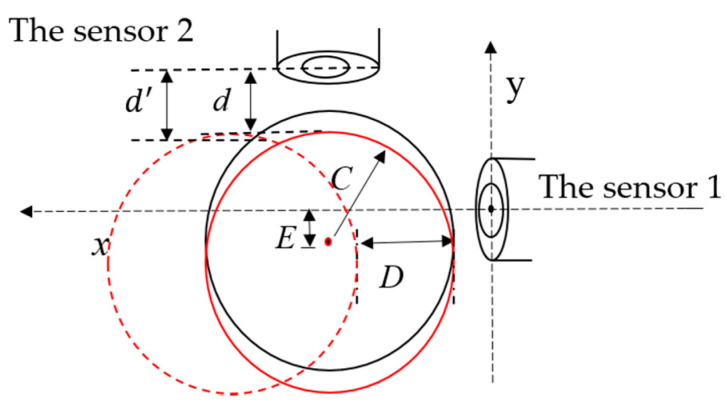
The orthogonal structure mathematical model.

In order to analyze the effect of the measured cylinder eccentricity in one direction on the output of the other sensor, a mathematical model diagram of the orthogonal structure was established, as shown in Figure 34. Assuming that the measured cylinder has an eccentricity in *Y* axis direction, and the measured cylinder is still moving in *X* axis direction, the output of the sensor 2 will inevitably change. The fluctuation of the sensor 2 output is directly related to whether the measured value of the sensor 2 can be regarded as the eccentric value of the sensor 1, especially when the displacement of the measured cylinder in both directions is very small. After the measured cylinder has an eccentricity in *Y* axis direction, *d* represents the distance from the sensor 2 to the measured cylinder. When the measured cylinder is moving in *X* axis direction, $d^{\prime }$ represents the distance from the sensor 2 to the measured cylinder.

From the geometrical relation:

(17) $$\Delta d=d^{\prime }-d=C-\sqrt{C^{2}-D^{2}}$$

**Figure 35:**
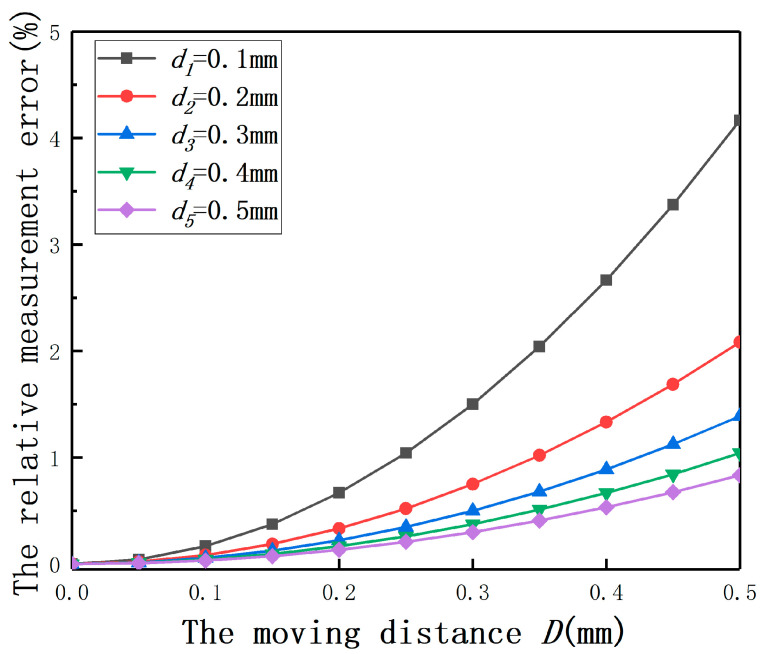
The relative measurement errors with the moving distance of the sensor 2.

If the fluctuation of the sensor 2 is defined as Δd/*d*, the relative measurement error is used to represent the fluctuation. The main influence on its control accuracy is the position detection accuracy of small displacement, so *d* is set as 0.1, 0.2, 0.3, 0.4, 0.5 mm and the range of *D* is 0–0.5 mm. As shown in Figure 35, the fluctuation of the sensor 2 output will not be very large and can be controlled within 4.5%. This means that the measurement value of the sensor 2 can be regarded as the eccentricity of sensor 1.

In the engineering application, when the rotor deviates during operation, the following situations will appear: (1) the output values of the two sensors are very small. (2) The output value of one sensor is large and the other is small. (3) The output values of the two sensors are very large.

**Figure 36:**
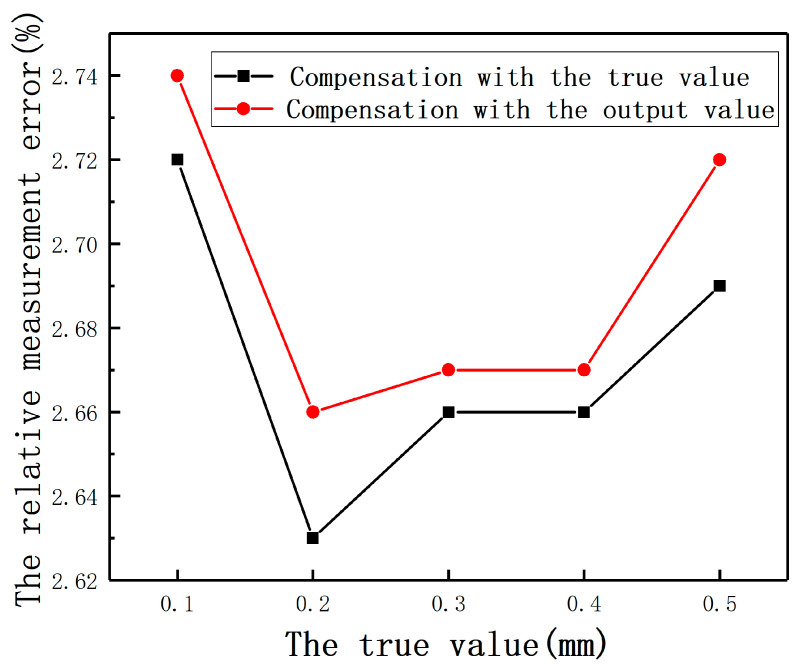
The relative measurement errors after compensation with the true value and the output value.

For the first case, based on the previous analysis, the output value can be approximately seen to be true when the eccentricity is very small, so the output value of the first sensor can be directly provided to the second sensor as the eccentricity. The output value of the second sensor after compensation is used as the eccentricity of the first sensor. By adjusting 3-dimensional freedom platforms, the measured cylinder was moved to the position with small *E* in both directions. *E* was set as 0.1, 0.2, 0.3, 0.4, and 0.5 mm, respectively. As shown in Figure 36, compared with the compensation effect of a known eccentricity, the error of using measured value instead of true value is acceptable.

**Figure 37:**
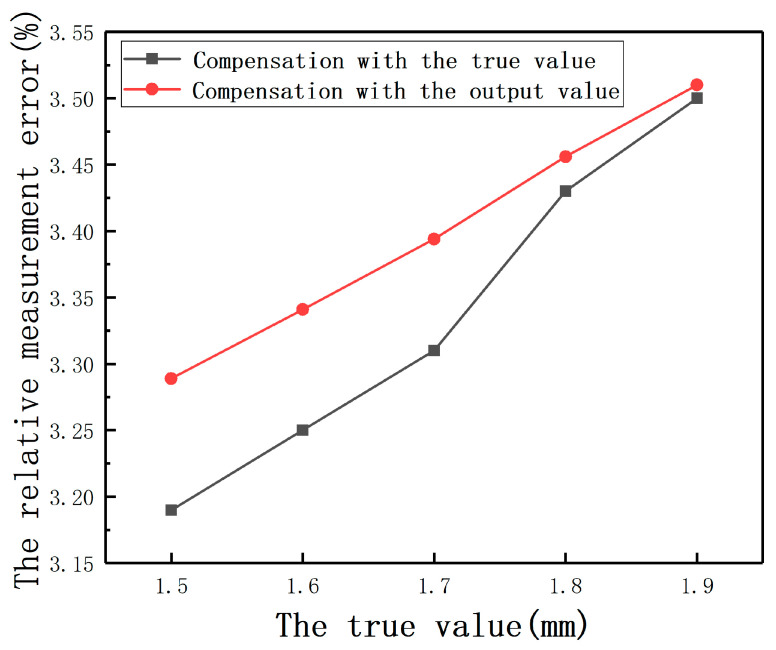
The relative measurement errors after compensation with the true value and the output value.

For the third case, according to the principle of closed-loop control, when the rotor is suspended in the equilibrium position, the main influence on its control accuracy is the position detection accuracy of small displacement. When the eccentricity of the rotor is large, the detection error mainly affects the dynamic performance of the control system. When the error is large, the speed of the control system is increased, which is acceptable for the control system. By adjusting 3-dimensional freedom platforms, in the direction of the first sensor probe, *E* was set as 1.5, 1.6, 1.7, 1.8, and 1.9 mm, respectively. In the direction of the second sensor probe, *E* was set as 1.5 mm. The output value of the second sensor can be directly provided to the first sensor as the eccentricity. As shown in Figure 37, compared with the compensation effect of a known eccentricity, the error of using measured value instead of true value is also acceptable.

**Figure 38:**
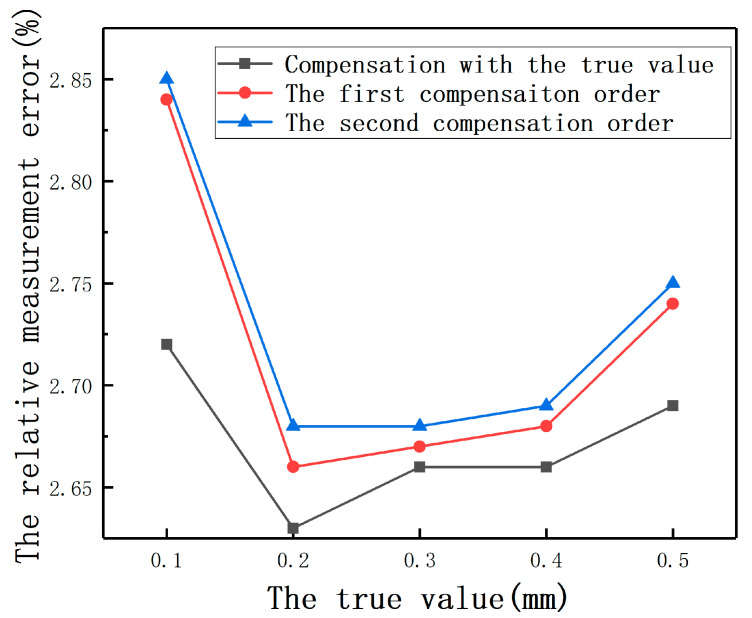
The relative measurement errors under different compensation sequences.

For the second case, the output value of one sensor is larger than another, which brings up a problem about the compensation order. To determine the compensation order, by adjusting 3-dimensional freedom platforms, in the direction of the first sensor, *E* was set as 0.1, 0.2, 0.3, 0.4, and 0.5 mm, respectively. In the direction of the second sensor, *E* was set as 1 mm. The first compensation order is that the small output value is used as the eccentricity of the large one. The output value after compensation is used as the eccentricity of the previous sensor. The second compensation order is contrary to the first one. The relative measurement error of the smaller true value is used as the standard of comparison. As shown in Figure 38, the relative measurement errors of the first compensation order are smaller than that of the second order.

**Figure 39:**
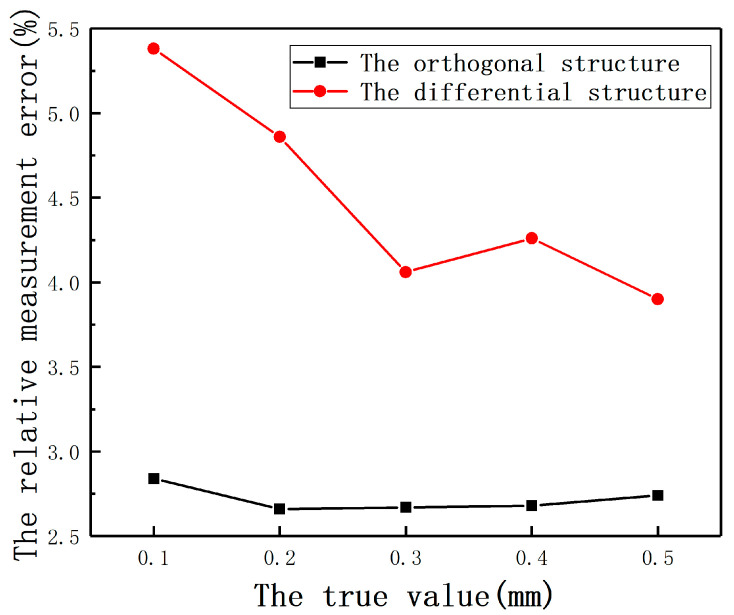
The relative measurement errors of the orthogonal structure and the differential structure.

In the eddy current displacement measurement, in order to reduce the influence of *E*, the differential structure is often used in the existing technology. Figure 39 shows the compensation effect of the compensation method based on the orthogonal structure that uses the measured value in the orthogonal direction as the true value of *E* and the hardware compensation effect of the differential structure. It can be seen from the result that the method proposed in this paper reduces the effect of *E* more significantly. Meanwhile, if the differential structure is used to detect the rotor radial position, the position detection system needs to be equipped with four sensors, which easily causes the electromagnetic interference between each other. However, the compensation method proposed in this section can be applied to the orthogonal structure, which only needs only two sensors. If four sensors are used in the position detection system, the redundancy and reliability of the system can be improved.

## 6. Conclusions

In this paper, the influence of eccentricity on the ECDS was analyzed by the FEM and the relative experiments. The following conclusions can be drawn:

(1) The existence of eccentricity has an appreciable influence on the measurement. Furthermore, the influence becomes smaller as the curvature increases.
(2) Compared with the single probe structure, the largest full-scale error under *E* = 2 mm is reduced by more than 40% after employing the differential structure.
(3) The largest full-scale error under *E* = 2 mm is less than 0.8% after compensation in the single probe structure, and 0.6% in the differential structure.
(4) To facilitate engineering application, the compensation method using the measured value instead of the true value was proposed, including the compensation order (the smaller value compensate for the lager value first).

Aiming at the eccentricity phenomena existing in the displacement measurement, this study mainly explored the static performance of the ECDS because the change of dynamic position was not considered. The future work will focus on the accuracy and the real-time capability of the position detection under the condition of the cylindrical specimen rotating and the position changing rapidly.
